# Gene Transfers Shaped the Evolution of De Novo NAD^+^ Biosynthesis in Eukaryotes

**DOI:** 10.1093/gbe/evu185

**Published:** 2014-08-27

**Authors:** Chad M. Ternes, Gerald Schönknecht

**Affiliations:** Department of Botany, Oklahoma State University

**Keywords:** horizontal gene transfer, endosymbiotic gene transfer, NAD^+^ biosynthesis, metabolism

## Abstract

NAD^+^ is an essential molecule for life, present in each living cell. It can function as an electron carrier or cofactor in redox biochemistry and energetics, and serves as substrate to generate the secondary messenger cyclic ADP ribose and nicotinic acid adenine dinucleotide phosphate. Although de novo NAD^+^ biosynthesis is essential, different metabolic pathways exist in different eukaryotic clades. The kynurenine pathway starting with tryptophan was most likely present in the last common ancestor of all eukaryotes, and is active in fungi and animals. The aspartate pathway, detected in most photosynthetic eukaryotes, was probably acquired from the cyanobacterial endosymbiont that gave rise to chloroplasts. An evolutionary analysis of enzymes catalyzing de novo NAD^+^ biosynthesis resulted in evolutionary trees incongruent with established organismal phylogeny, indicating numerous gene transfers. Endosymbiotic gene transfers probably introduced the aspartate pathway into eukaryotes and may have distributed it among different photosynthetic clades. In addition, several horizontal gene transfers substituted eukaryotic genes with bacterial orthologs. Although horizontal gene transfer is accepted as a key mechanism in prokaryotic evolution, it is supposed to be rare in eukaryotic evolution. The essential metabolic pathway of de novo NAD^+^ biosynthesis in eukaryotes was shaped by numerous gene transfers.

## Introduction

**Fig. 1.—:**
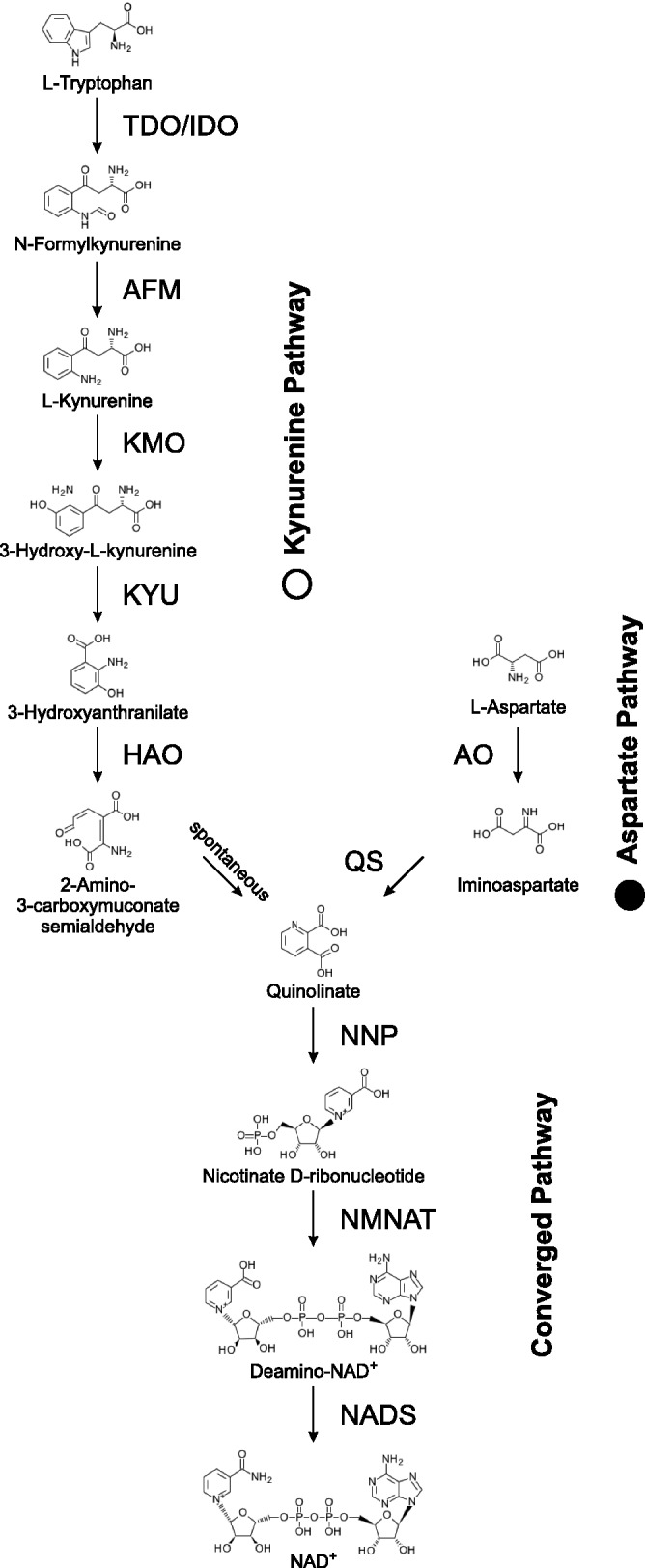
De novo NAD^+^ biosynthesis. The two different pathways for de novo NAD^+^ biosynthesis starting with either tryptophan for the kynurenine pathway or aspartate for the aspartate pathway are shown. Both pathways converge at quinolinate. Structural formulas for intermediates and all substrate and enzyme names according to KEGG (Kanehisa et al. 2014).

NAD^+^, nicotinamide adenine dinucleotide, is a coenzyme required in each living cell. As an electron carrier and cofactor of oxidoreductases NAD^+^ functions in redox biochemistry and energetic metabolism without being consumed. NAD^+^ is consumed in ADP-ribose transfer reactions, serves as a substrate for the synthesis of the Ca^2+^-mobilizing second messenger cyclic ADP ribose and nicotinic acid adenine dinucleotide phosphate, and functions as acceptor for protein lysine deacetylation (Belenky et al. 2007; Noctor et al. 2011; Pollak et al. 2007). These NAD^+^-consuming reactions require continuous de novo biosynthesis, which is described here. In addition, so-called salvage pathways, which will not be covered here, recycle components containing a nicotinamide ring (i.e., nicotinic acid, nicotinamide, nicotinamide ribose); these components result from NAD^+^ cleavage or are taken up with food (Katoh and Hashimoto 2004; Preiss and Handler 1958). All free-living eukaryotic organisms have the ability to synthesize NAD^+^ by one of two de novo pathways, the aspartate pathway (Griffith et al. 1975) or the kynurenine pathway starting with tryptophan (Gaertner and Subbayya Shetty 1977). Both pathways converge at quinolinate, which in three reaction steps is converted into NAD^+^ (fig. 1).

The kynurenine pathway consists of six reaction steps converting tryptophan into quinolinate. The first reaction step can be catalyzed by two different enzymes with different substrate specificity, whereas the last reaction step occurs spontaneously without the aid of an enzyme. The kynurenine pathway is characteristic for animals and fungi (Gaertner and Subbayya Shetty 1977; Rongvaux et al. 2003) and a few groups of Bacteria (Kurnasov et al. 2003; Lima et al. 2009). In mammals, about 90% of tryptophan is degraded through the kynurenine pathway, and some pathway intermediates represent neuroactive compounds (Rongvaux et al. 2003; Schwarcz et al. 2012). Yeast strains lacking one of the six enzymes of the kynurenine pathway require nicotinic acid for normal growth (Panozzo et al. 2002; Wogulis et al. 2008). The aspartate pathway converts aspartate into quinolinate in two enzyme-catalyzed reaction steps. This pathway is characteristic for Archaea, most groups of Bacteria (Begley et al. 2001; Griffith et al. 1975), as well as land plants and green algae (Viridiplantae) (Katoh et al. 2006; Lin et al. 2010). In *Arabidopsis thaliana* the two enzymes of the aspartate pathway are localized in plastids, and knock-out mutants lacking either of the two enzymes are embryo lethal (Katoh et al. 2006). In the unicellular green alga, *Chlamydomonas reinhardtii*, mutants lacking any of the five genes converting aspartate into NAD^+^ require supplemental nicotinamide (Lin et al. 2010).

**Fig. 2.—:**
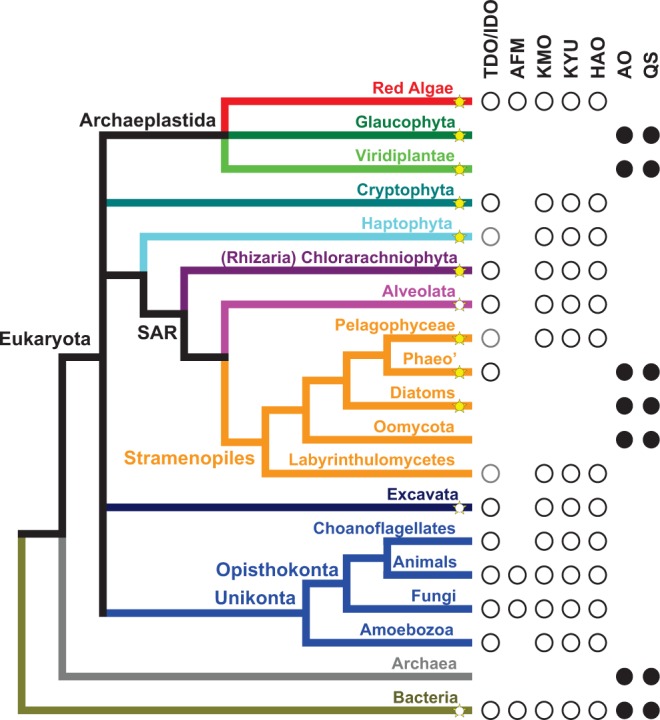
Phylogenetic distribution of de novo NAD^+^ biosynthesis pathways in eukaryotes shows a patchy distribution. Shown is the organismal phylogeny (Burki, Okamoto, et al. 2012; Keeling and Palmer 2008) for the major groups of life with emphasis on plastid bearing eukaryotes (star symbols; yellow, most species photoautotroph; white, some species photoautotroph; Phaeo’, Phaeophyceae or brown algae). For each clade, the presence of enzymes catalyzing the aspartate (closed symbols; AO, aspartate oxidase; QS, quinolinate synthase) or the kynurenine pathway (open symbols; TDO/IDO, tryptophan-/indoleamine 2,3-dioxygenase; AFM, arylformamidase; KMO, kynurenine 3-monooxygenase; KYU, kynureninase; HAO, 3-hydroxyanthranilate 3,4-dioxygenase) is indicated. Gray open symbols indicate that the IDO identified in this work, based on sequence similarity, might not actually catalyze the first reaction of the kynurenine pathway (see text). Most bacteria use the aspartate pathway, whereas some have the kynurenine pathway (see text). Very few bacteria, if any, seem to use both pathways in parallel.

Based on sequenced eukaryotic genomes, we compiled an overview of which de novo NAD^+^ biosynthesis pathway is present in different eukaryotic lineages. This resulted in an unexpected pattern for photosynthetic eukaryotes, with red algae (Rhodophyta) and the pelagophyte *Aureococcus anophagefferens* using the kynurenine pathway, whereas land plants and green algae (Viridiplantae), diatoms, and brown algae (Phaeophyta) appear to use the aspartate pathway for NAD^+^ de novo biosynthesis (fig. 2). A phylogenetic analysis indicates that the evolution of de novo NAD^+^ biosynthesis was driven by several instances of gene transfer, both endosymbiotic gene transfer and horizontal gene transfer from bacteria or archaea into eukaryotic genomes.

## Materials and Methods

Homologous protein sequences were identified by BLAST (Altschul et al. 1997) searches at National Center for Biotechnology Information (NCBI) (Pruitt et al. 2007), KEGG (Kanehisa et al. 2014), http://genome.jgi.doe.gov/, http://www.broadinstitute.org, http://cyanophora.rutgers.edu/cyanophora/home.php (last accessed September 5, 2014), and collected using MEGA 5 (Tamura et al. 2007). BLAST runs were carried out separately for different clades for broad phylogenomic sampling. Incomplete or highly redundant sequences (no pair of sequences with >90% amino acid identity was allowed) were removed. Multiple sequence alignments were generated with T-Coffee (Notredame et al. 2000) using the “accurate” mode, which combines alignment using structural information with alignment using homology extension (Di Tommaso et al. 2011). High scoring portions of multiple sequence alignments (T-Coffee score 5–9, deviations mentioned in figure legend) were extracted (Talavera and Castresana 2007). Alignments are available through TreeBase (http://purl.org/phylo/treebase/phylows/study/TB2:S15669, last accessed September 5, 2014). An estimation which model of protein evolution best fits the multiple sequence alignment was generated by ProtTest (Abascal et al. 2005). The best model, in most cases LG+I+G (otherwise specified in figure legend), was used to generate phylogenetic trees with PhyML 3.0 (Guindon et al. 2010) estimating branch support values by nonparametric bootstrap with 200 replicates. The LG model of protein evolution is not implemented in the MPI version of MrBayes 3.1.2 (Altekar et al. 2004), and we used the best model implemented, for all but one multiple sequence alignment (specified in figure legend) WAG+I+G. MrBayes was run with 12 chains at a temperature of 0.05 for 5,000,000 generations sampling every 100th generation; the first 25% of samples were ignored when calculating parameters and consensus tree. Trace files from Bayesian MCMC runs were inspected with Tracer 1.6 (http://beast.bio.ed.ac.uk/Tracer, last accessed September 5, 2014) to ensure that likelihoods beyond the 25% cutoff where stable and showed an approximately Gaussian distribution. Displayed are unrooted Bayesian trees with larger clades collapsed (full trees are available at TreeBase; http://purl.org/phylo/treebase/phylows/study/TB2:S15669, last accessed September 5, 2014); only support values >50% are given.

Alternative evolutionary models, with specific clades constrained as monophyletic, were tested using likelihood values. Constrained and unconstrained Bayesian trees were inferred using MrBayes v3.1.2, and the harmonic mean (*H*) and Akaike’s information criterion through Markov chain Monte Carlo (AICM) (Baele et al. 2012) of log-likelihood values from the stationary phase of Bayesian analyses were calculated by Tracer 1.6. Differences between harmonic means (Δ*H*) and differences between AICM values (ΔAICM) usually showed a ratio close to Δ*H* ≈ ΔAICM/−2, as expected. A constraint tree was regarded significantly different from the unconstrained tree when Δ*H* > 3 SD (SD, standard deviation of log-likelihood values, ranging from 10.6 to 16; using the larger SD), and −ΔAICM > 6 SD. Constrained and unconstrained maximum-likelihood trees were inferred using RAxML 8.0 (Stamatakis 2014), and calculated site-likelihood values were used as input for CONSEL (Shimodaira and Hasegawa 2001) to calculate *P* values of the Kishino–Hasegwa test and the approximate unbiased test (Shimodaira 2002) (supplementary table S1, Supplementary Material online). At *P* values <5%, log-likelihood values of constraint trees were regarded as significantly lower.

## Results

### Patchy Distribution of Kynurenine and Aspartate Pathways

In eukaryotes, the two de novo NAD^+^ biosynthesis pathways seem to be mutually exclusive (fig. 2). So far, no eukaryotic organism has been discovered that contains both pathways. Earlier reports (Katoh and Hashimoto 2004) of the existence of enzymes for the kynurenine pathway in rice were not corroborated by our bioinformatics and phylogenetic analyses. Some parasitic eukaryotes, such as *Plasmodium*, lack de novo NAD^+^ biosynthesis and only operate salvage pathways taking up nicotinic acid or nicotinamide from their hosts (Plata et al. 2010). In Eukaryota the kynurenine pathway is observed in most clades, with the exception of some photosynthetic clades (or clades closely related to photosynthetic ones), which use the aspartate pathway for quinolinate biosynthesis (fig. 2). This phylogenetic distribution of the two different de novo NAD^+^ biosynthesis pathways indicates that the kynurenine pathway is the ancestral pathway present in the last common ancestor of all eukaryotes. Endosymbiosis giving rise to photosynthesis in some eukaryotic lineages was probably driving the substitution of the kynurenine pathway by the aspartate pathway. Photosynthesis in eukaryotes is thought to have originated from an endosymbiosis event, when a nonphotosynthetic eukaryotic cell took up a photosynthetic cyanobacterium, which evolved into an endosymbiont giving rise to the chloroplast (Keeling 2010; Reyes-Prieto et al. 2007). Besides the chromatophores in the filose amoeba *Paulinella chromatophora*, which result from a more recent endosymbiosis event (Marin et al. 2005), all plastids in eukaryotes go back to a single ancient endosymbiosis event. The evolution of endosymbiosis was accompanied by massive gene transfer from the cyanobacterial genome into the nucleus of the eukaryotic host cell. Bioinformatic analyses indicate that thousands of genes in land plant genomes originate from this endosymbiotic gene transfer from a cyanobacterial genome (Dagan et al. 2013; Timmis et al. 2004). As the aspartate pathway is only observed in plastid-bearing eukaryotes, or in lineages which probably had a plastid-bearing ancestor, it seems reasonable to assume that the aspartate pathway was introduced into eukaryotes by endosymbiotic gene transfer from a cyanobacterial genome.

Primary endosymbiosis of a cyanobacterium gave rise to the Archaeplastida including Rhodophyta (red algae), Glaucophyta, and Viridiplantae (green algae and land plants) (fig. 2). For Viridiplantae the presence of the aspartate pathway (and absence of the kynurenine pathway) is well established, and the similarity of the two enzymes converting aspartate into quinolinate to orthologous bacterial enzymes has been reported (Katoh et al. 2006; Noctor et al. 2011). Our analysis of the recently published genome of the glaucophyte *Cyanophora paradoxa* (Price et al. 2012) indicates the presence of the aspartate pathway (see below). In contrast, the third clade of the Archaeplastida, the red algae (Rhodophyta), uses the kynurenine pathway to synthesize quinolinate from tryptophan. All five red algal genomes, from *Cyanidioschyzon merolae* (Matsuzaki et al. 2004), *Galdieria sulphuraria* (Schönknecht et al. 2013), *Pyropia yezoensis* (Nakamura et al. 2013), *Chondrus crispus* (Collén et al. 2013), and *Porphyridium purpureum* (Bhattacharya et al. 2013) appear to encode the kynurenine pathway, while lacking the aspartate pathway.

Stramenopiles, which include several photosynthetic clades, such as diatoms and brown algae, are thought to have acquired photosynthetic capacity by a secondary endosymbiosis following the uptake of a unicellular red alga (Baurain et al. 2010; Cavalier-Smith 1999). Somewhat unexpected diatoms, brown algae, and Oomycota (water molds or downy mildew) appear to use the aspartate pathway for quinolinate biosynthesis, and not the conserved kynurenine pathway observed in red algae and some other Stramenopile clades (Labyrinthulomycetes and Pelagophyceae; fig. 2). This raises the question how diatoms, brown algae, and Oomycota might have acquired the aspartate pathway? They could have acquired it from green algae. Thousands of genes of potential green algal origin have been detected in diatoms, giving rise to the hypothesis that Stramenopiles have undergone a cryptic secondary endosymbiosis with a green alga before acquiring the red algal endosymbiont (Moustafa et al. 2009). However, this hypothesis has been criticized, and it has been questioned how many genes of green algal origin can actually be detected in diatoms (Burki, Flegontov, et al. 2012; Deschamps and Moreira 2012; Woehle et al. 2011). Within Stramenopiles the basic clade of Labyrinthulomycetes encodes enzymes for the kynurenine pathway, indicating that this lineage might have split off from other Stramenopiles before the aspartate pathways were acquired. Surprisingly, the genome of the pelagophyte *Au. anophagefferens* (Gobler et al. 2011) lacks genes for the aspartate pathway but encodes genes for the kynurenine pathway, in contrast to other photosynthetic Stramenopiles (fig. 2), which can probably not be explained by conservation of the kynurenine pathway (see below). The presence of the kynurenine pathway in Cryptophyta and Haptophyta, which also acquired plastids by secondary endosymbiosis with a red alga, can probably be best explained by conservation of the kynurenine pathway in the host (fig. 2).

To develop a better understanding why some photosynthetic eukaryotes use the aspartate pathway whereas others use the kynurenine pathway for quinolinate biosynthesis, an evolutionary analysis of the enzymes catalyzing NAD^+^ de novo biosynthesis was performed.

### Evolutionary Analysis of Aspartate Pathway Enzymes

**Fig. 3.—:**
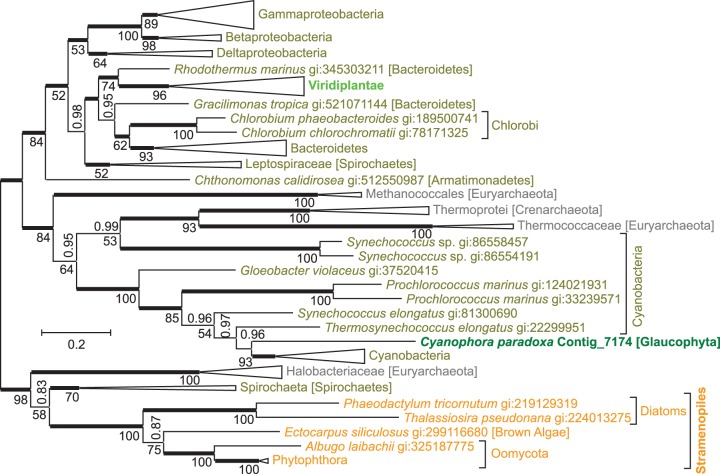
An evolutionary tree for l-aspartate oxidase indicates separate origins of eukaryotic sequences. The unrooted Bayesian tree shows posterior probabilities above the branches and PhyML bootstrap values displayed as percentages below the branches. Thickened horizontal lines represent 1.0 Bayesian posterior probability. Trees with significantly lower log-likelihoods were obtained when monophyly was enforced for all sequences from Eukaryota, from any two of the three eukaryotic branches (Viridiplantae, Glaucophyta, Stramenopiles), or from Bacteria (supplementary table S1, Supplementary Material online). Larger clades have been collapsed for presentation and size is indicative of the number of taxa within the clade (full trees are available at TreeBase; http://purl.org/phylo/treebase/phylows/study/TB2:S15669, last accessed September 5, 2014). Scale bar represents 0.2 substitutions per site.

The aspartate pathway consists of two reaction steps converting aspartate into quinolinate, the precursor at which both de novo NAD^+^ biosynthesis pathways converge (fig. 1). **l-aspartate oxidase** (EC 1.4.3.16) catalyzes the first step, the reaction of aspartate with oxygen to form iminoasparate. An evolutionary tree of aspartate oxidases (fig. 3) shows the enzyme from *C. paradoxa* as monophyletic with cyanobacterial aspartate oxidases as expected for a gene that was acquired through endosymbiotic gene transfer from a cyanobacterial endosymbiont. The protein sequence from *C. paradoxa* resides deep within a cyanobacterial branch, next to a sequence from *Thermosynechococcus elongates* BP-1, close to the position where plastids were supposed to branch from the cyanobacterial tree according to Criscuolo and Gribaldo (2011). However, aspartate oxidases from Viridiplantae and Stramenopiles show a different evolutionary origin and are not closely related to cyanobacterial sequences. Instead, sequences from Viridiplantae form a monophyletic group with Bacteroidetes, whereas Stramenopile oxidases are monophyletic with spirochaetes and salt-loving, aquatic archaea. Surprisingly, aspartate oxidases from three different clades of photosynthetic eukaryotes seem to originate from three different lineages within Bacteria or Archaea. For all three eukaryotic clades, monophyly with a different prokaryotic group is well supported by Bayesian posterior probabilities and bootstrap support values. Alternative evolutionary trees with topological constrains enforcing monophyly of any two of the three eukaryotic photosynthetic clades had extremely low probabilities (supplementary table S1, Supplementary Material online).

**Fig. 4.—:**
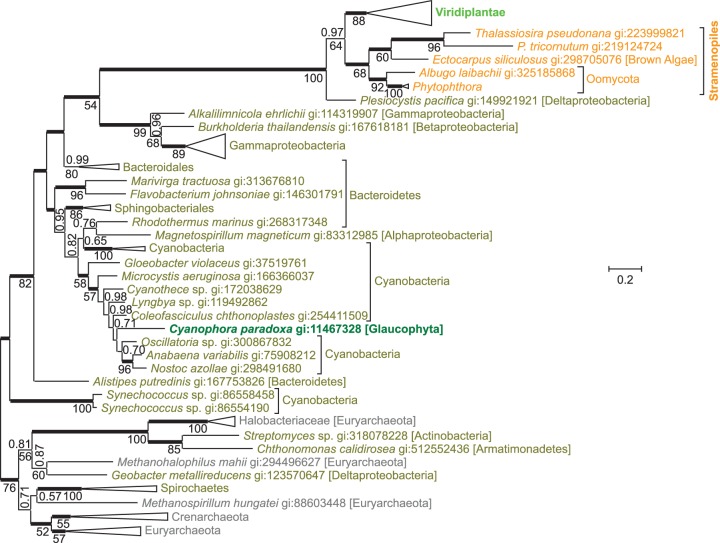
An evolutionary tree for quinolinate synthase indicates separate origins of eukaryotic sequences. The unrooted Bayesian tree shows posterior probabilities above the branches and PhyML bootstrap values below the branches. Thickened horizontal lines represent 1.0 Bayesian posterior probability. Trees with significantly lower log-likelihoods were obtained when monophyly was enforced for all sequences from Eukaryota, from Viridiplantae plus *Cyanophora paradoxa*, from Bacteria, or from Archaea (supplementary table S1, Supplementary Material online).

The second reaction step of the aspartate pathway is catalyzed by **quinolinate synthase** (EC 2.5.1.72). Iminoasparate reacts with dihydroxyacetone phosphate to generate quinolinate. An evolutionary tree of quinolinate synthases (fig. 4) shows the sequence from *C. paradoxa* deep within a cyanobacterial branch, in agreement with an origin by endosymbiotic gene transfer. The gene for quinolinate synthase is part of the cyanelle genome of *C. paradoxa*, further supporting its endosymbiotic origin. Quinolinate synthases from Stramenopiles and Viridiplantae are not closely related to cyanobacterial sequences, but form a separate monophyletic group together with a synthase from the proteobacterium *Plesiocystis pacifica*. This separate monophyletic group as well as the descent of *C. paradoxa* quinolinate synthase from cyanobacterial sequences is well supported by Bayesian posterior probability and bootstrap support values. Alternative trees with topological constraints enforcing monophyly of all three photosynthetic eukaryotic clades had extremely low probabilities (supplementary table S1, Supplementary Material online). Quinolinate synthase or aspartate oxidase from *Ar. thaliana* has been shown to complement *Escherichia coli* mutants deficient in these enzymes, indicating functional similarity between higher plant and bacterial orthologs (Katoh et al. 2006).

It appears that aspartate oxidase and quinolinate synthase have different evolutionary origins in different photosynthetic eukaryotic clades. To explain this somewhat complex evolutionary pattern, several separate gene transfer events have to be postulated. The high similarity of both enzymes of the aspartate pathway between the glaucophyte *C. paradoxa* and cyanobacteria seems to support acquisition of the aspartate pathway by photosynthetic eukaryotes through endosymbiotic gene transfer from the cyanobacterial endosymbiont. When the three lineages of Archaeplastida (namely Glaucophyta, red algae, and green plants) split, Glaucophyta seem to have kept the cyanobacterial genes encoding aspartate oxidase and quinolinate synthase, whereas the ancestor of green plants seems to have substituted the cyanobacterial genes through horizontal gene transfer with genes from nonphotosynthetic bacteria. In the lineage giving rise to red algae, the aspartate pathway was not established, and the kynurenine pathway remained active. The monophyly of quinolinate synthases from green plants and Stramenopiles indicates horizontal gene transfer between green plant and Stramenopiles. The close similarity of Stramenopile aspartate oxidases to prokaryotic aspartate oxidases could be explained by another horizontal gene transfer from a spirochaete or a halobacterium into an ancestor of Stramenopiles.

In higher plants it has been demonstrated that aspartate oxidase and quinolinate synthase are located in plastids, in agreement with their endosymbiotic origin (Katoh et al. 2006). Somewhat unexpected, nicotinate-nucleotide pyrophosphorylase of higher plants is targeted to plastids as well (Katoh et al. 2006).

### Evolutionary Analysis of Converged Pathway Enzymes

**Fig. 5.—:**
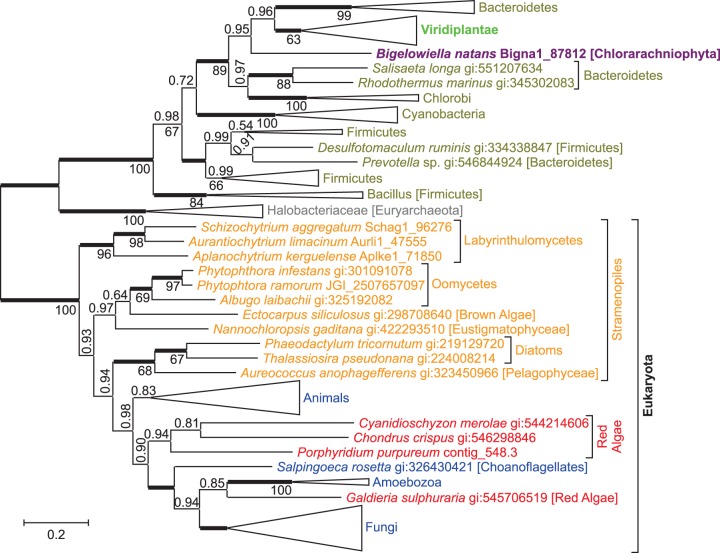
An evolutionary tree for nicotinate-nucleotide pyrophosphorylase indicates that some photosynthetic eukaryotes acquired genes through transfers. The unrooted Bayesian tree shows posterior probabilities above the branches and PhyML bootstrap values below the branches. Thickened horizontal lines represent 1.0 Bayesian posterior probability. A tree with significantly lower log-likelihood was obtained when monophyly was enforced for all sequences from Eukaryota (supplementary table S1, Supplementary Material online). A tree with practically identical log-likelihood was obtained when monophyly was enforced for all sequences from Viridiplantae plus *Bigelowiella natans* (form a monophyletic clade within Bacteroidetes in PhyML tree). The sequence from *Cyanophora paradoxa* was omitted from an evolutionary analysis because it is incomplete. The best BLASTp hit for this incomplete sequence in the nr database from NCBI was with a nicotinate-nucleotide pyrophosphorylase sequence from *Capsaspora owczarzaki* (167 score, 67% coverage), and all top 100 BLASTp hits were with sequences from nonphotosynthetic eukaryotes, indicating possible descent of nicotinate-nucleotide pyrophosphorylase in Glaucophyta from the last common ancestor of eukaryotes.

**Nicotinate-nucleotide pyrophosphorylase** (EC 2.4.2.19) catalyzes the reaction of quinolinate with 5-phosphoribosyl diphosphate to synthesize nicotinate d-ribonucleotide plus pyrophosphate and CO_2_ (fig. 1). As the aspartate pathway and kynurenine pathway converge at quinolinate, all free-living eukaryotes contain nicotinate-nucleotide pyrophosphorylase. In an evolutionary tree for this enzyme eukaryotic sequences form one monophyletic branch, with the exception of sequences from green plants (Viridiplantae) and *Bigelowiella natans*, which reside together deep within the bacterial branch (fig. 5). Nicotinate-nucleotide pyrophosphorylases in most eukaryotic lineages appear to descent from the last common ancestor of all eukaryotes. In contrast, nicotinate-nucleotide pyrophosphorylases from Viridiplantae form a sister group to sequences from Bacteroidetes, indicating that the nicotinate-nucleotide pyrophosphorylase gene of eukaryotic origin was substituted by horizontal gene transfer from a bacterium in an early ancestor of Viridiplantae. Alternative trees enforcing monophyly of all eukaryotic sequences had low probabilities (supplementary table S1, Supplementary Material online). The nicotinate-nucleotide pyrophosphorylase gene from tobacco has been shown to functionally complement *Es. coli* cells lacking the orthologous enzyme (Sinclair et al. 2000) emphasizing the similarity between higher plant and bacterial orthologs.

The similarity of nicotinate-nucleotide pyrophosphorylase from *B. natans* and green plants can probably be explained by endosymbiotic gene transfer. The chlorarachniophyte *B. natans* is known to have acquired its plastid through secondary endosymbiosis with an ancient green alga (Archibald et al. 2003). Endosymbiotic gene transfer from an ancestral green algal endosymbiont into the genome of *B. natans* probably resulted in the substitution of the eukaryotic pyrophosphorylase gene.

**Nicotinate-nucleotide adenylyltransferase** catalyzes the conversion of ATP plus beta-nicotinate d-ribonucleotide into deamido-NAD^+^ plus diphosphate (EC 2.7.7.18). This enzyme also catalyzes the conversion of ATP plus nicotinamide d-ribonucleotide into NAD^+^ plus diphosphate (nicotinamide-nucleotide adenylyltransferase; EC 2.7.7.1). In an evolutionary tree of nicotinate-nucleotide adenylyltransferases (supplementary fig. S1, Supplementary Material online), eukaryotic sequences form one monophyletic branch and bacterial sequences form another branch. The topology of the eukaryotic branch does not properly reflect established eukaryotic phylogeny (compare fig. 2). Enforcing monophyly of established eukaryotic clades does result in alternative trees with almost identical log-likelihood values (supplementary table S1, Supplementary Material online).

The final step in de novo NAD^+^ biosynthesis is catalyzed by **NAD^+^ synthase** (EC 6.3.5.1) catalyzing the conversion of deamino-NAD^+^ into NAD^+^ using glutamine as NH_3_ donor (fig. 1). An evolutionary tree of NAD^+^ synthases shows all eukaryotic sequences, with exception of the one from *Au. anophagefferens*, as a monophyletic group (supplementary fig. S2, Supplementary Material online). The NAD^+^ synthase sequence from *Au. anophagefferens* resides within a branch composed entirely of bacterial protein sequences, suggesting horizontal gene transfer from a bacterium. An alternative tree enforcing monophyly of all eukaryotic sequences had extremely low probability (supplementary table S1, Supplementary Material online).

### Evolutionary Analysis of Kynurenine Pathway Enzymes

Given the unexpected number of horizontal or endosymbiotic gene transfers in the aspartate pathway and the converged pathway, we conducted evolutionary analyses for kynurenine pathway enzymes. The first reaction in the kynurenine pathway produces *N*-formylkynurenine from tryptophan and molecular oxygen. This reaction is catalyzed by either the highly specific tryptophan 2,3-dioxygenase (EC:1.13.11.11) or the less specific indoleamine 2,3-dioxygenase (EC:1.13.11.52). An evolutionary tree of **tryptophan 2,3-dioxygenase** does not show the expected separation of eukaryotic and bacterial protein sequences (supplementary fig. S3, Supplementary Material online), and alternative trees enforcing monophyly of all eukaryotic or all bacterial sequences have low probability (supplementary table S1, Supplementary Material online). The sequence from *Naegleria gruberi* is the only eukaryotic protein sequence in a prokaryotic branch, probably indicating horizontal gene transfer of a bacterial tryptophan 2,3-dioxygenase gene into (an ancestor of) *N. gruberi*. The tryptophan 2,3-dioxygenases from *Perkinsus marinus* and *Guillardia theta* might originate from horizontal gene transfer from bacteria, or bacteria might have acquired eukaryotic genes, followed by gene transfers between bacteria. The evolutionary analysis of tryptophan 2,3-dioxygenases is limited by relative short multiple sequence alignments (250–350 amino acids) and limited sequence availability (some eukaryotic clades only contain indoleamine 2,3-dioxygenase).

An evolutionary tree of **indoleamine 2,3-dioxygenase** (supplementary fig. S4, Supplementary Material online) has three main branches, all three containing sequences from both Eukaryota and Bacteria. Alternative trees enforcing monophyly of all eukaryotic or all bacterial sequences have low probability (supplementary table S1, Supplementary Material online). However, only enzymes in one of the three main branches seem to catalyze the reaction of tryptophan and O_2_ with an affinity and at a rate expected for effective tryptophan degradation. This is the branch containing sequences from mammals, fungi, and the bacteria *Gemmatimonas aurantiaca* and *Streptomyces scabiei* (Yuasa et al. 2011). Enzymes from the other two branches that were biochemically characterized show very low reaction rates, questioning a function in tryptophan degradation. These are fungal indoleamine 2,3-dioxygenases γ (Yuasa and Ball 2012) and recombinant indoleamine 2,3-dioxygenases from *Erythrobacter litoralis* (Alphaproeobacteria) and *Neptuniibacter caesariensis* (Gammaproteobacteria) (Yuasa et al. 2011). On the other hand, indoleamine 2,3-dioxygenases from the stramenopiles *Aplanochytrium kerguelense*, *Aurantiochytrium limacinum*, *Schizochytrium aggregatum* (Labyrinthulomycetes), and *Au. anophagefferens* (Pelagophyceae), and the haptophyte *Emiliania huxleyi* located in these two branches were the only candidates identified in our BLAST searches that seem suitable to catalyze the reaction with tryptophan. It seems possible that for some stramenopiles and haptophyta the first enzyme of the kynurenine pathway still awaits identification (indicated by gray open circles in fig. 2).

In the second reaction step of the kynurenine pathway, *N*-formylkynurenine is hydrolyzed into l-kynurenine and formate. This reaction can be catalyzed by two different, nonorthologous enzymes. In many eukaryotes, an α/β hydrolase fold enzyme with an esterase/lipase domain functions as **arylformamidase** (EC 3.5.1.9) (Pabarcus and Casida 2002; Wogulis et al. 2008), whereas in many bacteria a cyclase functions as arylformamidase (Kurnasov et al. 2003). Eukaryotic arylformamidases show little conservation, with just 22% amino acid similarity (11% identity) between yeast and mouse (Wogulis et al. 2008). Outside Opisthokonta hardly any eukaryotic arylformamidase has been annotated, and BLAST searches with opisthokont sequences do not result in meaningful hits outside Opisthokonta, preventing an evolutionary analysis of eukaryotic arylformamidases. In the genome of the thermoacidophilic red alga *G. sulphuraria* a cyclase with homology to bacterial arylformamidases has been annotated, and an evolutionary analysis indicates that *G. sulphuraria* acquired this enzyme through horizontal gene transfer from Actinobacteria (Schönknecht et al. 2014).

**Fig. 6.—:**
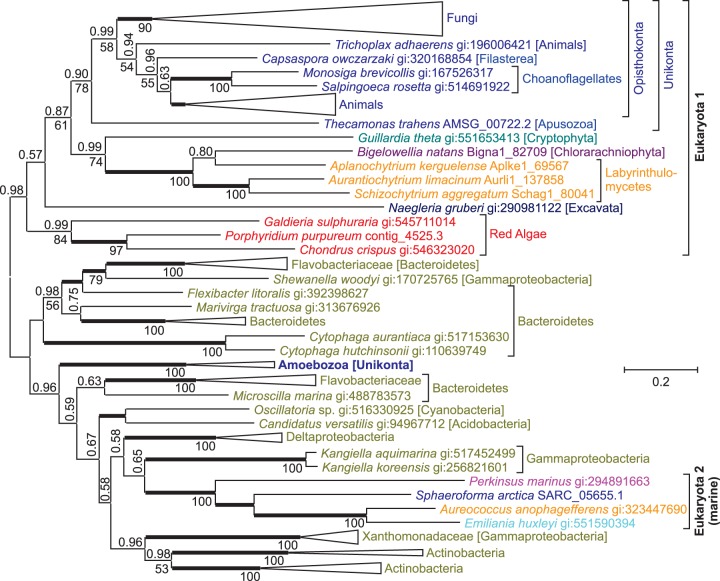
An evolutionary tree for kynurenine-3-monooxygenase indicates that some unicellular eukaryotes acquired genes through horizontal transfers. The unrooted Bayesian tree shows posterior probabilities above the branches and PhyML bootstrap values below the branches. Thickened horizontal lines represent 1.0 Bayesian posterior probability. Trees with significantly lower log-likelihood were obtained when monophyly was enforced for all sequences from eukaryotes, from Amoebozoa plus other Unikonta (without *Sphaeroforma arctica*), from *S. arctica* plus other Opisthokonta, or from *Emiliania huxleyi*, *Aureococcus anophagefferens*, and *Perkinsus marinus* plus Labyrinthulomycetes and Chlorarachniophyta (i.e., SAR and Haptophyta; fig. 2 and supplementary table S1, Supplementary Material online).

**Kynurenine-3-monooxygenase** (EC:1.14.13.9) catalyzes the third reaction step of the kynurenine pathway, the hydroxylation of l-kynurenine into 3-hydroxy-l-kynurenine. In an evolutionary tree of this enzyme, sequences from most eukaryotic clades form one major branch (fig. 6). The other major branch, with bacterial sequences, however, has several eukaryotic sequences interspersed, indicating possible instances of horizontal gene transfer. Alternative evolutionary trees enforcing monophyly of eukaryotic clades have low probability (supplementary table S1, Supplementary Material online). Earlier phylogenetic analyses of kynurenine-3-monooxygenases also place the sequence from *Dictyostelium discoideum* (Amoebozoa) in one branch with bacterial sequences (Lima et al. 2009), in good agreement with our results. Phylogenomic analyses for the amoebozoans *D. discoideum* (Andersson 2011; Eichinger et al. 2005) and *Entamoeba histolytica* (Loftus et al. 2005) identified several instances of horizontal gene transfer from bacterial genomes, and it has been suggested that the phagotrophic life style of amoebozoa promoted DNA uptake (Doolittle 1998). Interestingly, none of the three phylogenomic studies with amoebozoa seem to have identified kynurenine-3-monooxygenase as a candidate for horizontal gene transfer. This might be caused by several interspersed eukaryotic sequences rather than just one, making it harder to detect a specific horizontal gene transfer event.

Somewhat surprising is a branch with sequences from four eukaryotes from different lineages embedded in the bacterial branch (“Eukaryota 2 [marine]” in fig. 6). *Aureococcus anophagefferens* and *P. marinus* both belong to the SAR (Stramenopiles, Alveolata, and Rhizaria) supergroup, which may have a common ancestor with the haptophyte *Em. huxleyi* (Read et al. 2013) (fig. 2). However, sequences from other stramenopiles (Labyrinthulomycetes) and from *B. natans* (Rhiziaria) are not monophyletic with the sequences from *Au. anophagefferens*, *P. marinus*, and *Em. huxleyi* (supplementary table S1, Supplementary Material online), indicating that the latter three probably acquired their kynurenine-3-monooxygenase through horizontal gene transfer. Moreover, the sequence from *Sphaeroforma arctica* (Ichthyosporea, Opisthokonta) is not monophyletic with other Opisthokont sequences (fig. 6 and supplementary table S1, Supplementary Material online) and was probably acquired through horizontal gene transfer as well. Although phylogenetically distant, *Au. anophagefferens*, *P. marinus*, *Em. huxleyi*, and *S. arctica* are marine organisms, all four occur in the North Atlantic. The sequences next to this eukaryotic branch are from *Kangiella koreensis* and *Kangiella aquimarina*, marine bacteria from the family of Alcanivoracaceae, with several species in the North Atlantic. The co-occurrence of homologous proteins in distantly related organisms within the same environments has been observed before in studies of horizontal gene transfer both in prokaryotes (Beiko et al. 2005) and in eukaryotes (Andersson 2011).

**Kynureninase** (EC:3.7.1.3) catalyzes the fourth step of the kynurenine pathway, the hydrolysis of 3-hydroxy-l-kynurenine into 3-hydroxyanthranilate and alanine, and **3-hydroxyanthranilate 3,4-dioxygenase** (EC:1.13.11.6) catalyzes the fifth step, the decyclization of 3-hydroxyanthranilate into 2-amino-3-carboxymuconate semialdehyde (fig. 1). For both enzymes evolutionary trees have two major branches, one with eukaryotic and the other with bacterial sequences (supplementary figs. S5 and S6, Supplementary Material online). Earlier evolutionary analyses of kynureninases and 3-hydroxyanthranilate 3,4-dioxygenases were limited to Unikonta, but had a wider coverage of bacterial sequences (Lima et al. 2009), and resulted in comparable tree topologies.

Genes that have been acquired through horizontal gene transfer from bacteria or archaea often have no or fewer introns and can differ in GC content compared with the complete genome. For the three NAD^+^ biosynthesis genes in photosynthetic eukaryotes that were probably acquired through horizontal gene transfer from bacteria (namely, aspartate oxidase gene, quinolinate synthase gene, and nicotinate-nucleotide pyrophosphorylase gene), no significant reduction in intron number or deviation in GC content could be detected. This is not entirely unexpected given that most of the gene transfer events described here probably occurred several hundreds of million years ago, providing ample amount of time for gene signatures to become indistinguishable from the rest of the genome.

## Discussion

**Fig. 7.—:**
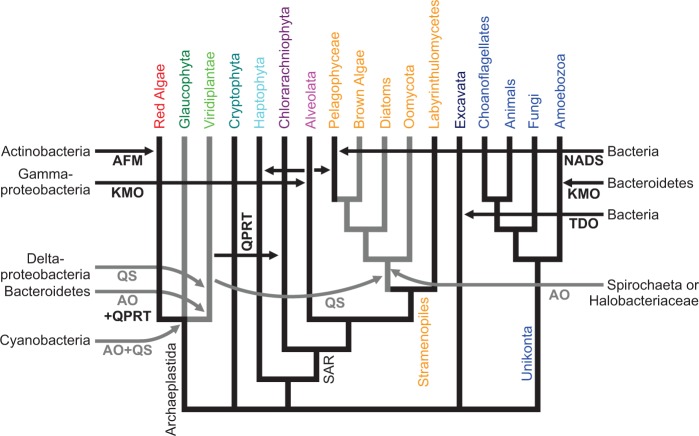
Gene transfers in the evolution of de novo NAD^+^ synthesis. Shown is an organismal phylogeny (Burki, Okamoto, et al. 2012; Keeling and Palmer 2008) of the major eukaryotic lineages with emphasis on plastid bearing clades. Horizontal arrows indicate putative horizontal gene transfers from Bacteria or Archaea, with the most likely “donor clade” indicated. The horizontal gene transfer of a KMO gene from a proteobacterium into *Sphaeroforma arctica* (Ichthyosporea, Opisthokonta) is omitted for clarity. Abbreviated enzymes names are AFM, arylformamidase; AO, aspartate oxidase; KMO, kynurenine 3-monooxygenase; NADS, NAD^+^ synthase; QPRT, nicotinate-nucleotide pyrophosphorylase (quinolinate phosphoribosyltransferase); QS, quinolinate synthase; SAR, Stramenopiles, Alveolates, and Rhizaria; TDO, tryptophan dioxygenase. Black indicates kynurenine (and converged) pathway, gray indicates aspartate pathway.

In figure 7, the different gene transfers contributing to the evolution of de novo NAD^+^ biosynthesis are summarized. The gene transfer events displayed are the result of rather conservative evolutionary analyses. Only transfer events with good statistical support, both from Bayesian (Δ*H* > 3 SD and −ΔAICM > 6 SD) and maximum-likelihood analyses (*P* values < 5%), were included. We concentrated on transfer events from bacteria or archaea into eukaryotic genomes. Possible transfer events of indoleamine dioxygenase homologs (supplementary fig. S4, Supplementary Material online) were not included, as these enzymes may not actually catalyze tryptophan degradation (see above).

### Evolutionary Origin of the Aspartate Pathway in Eukaryotes

To explain the current phylogenetic pattern of aspartate oxidases and quinolinate synthases in eukaryotes, a minimum of five gene transfer events have to be postulated. 1) Both genes were probably introduced into eukaryotes by endosymbiotic gene transfer from the cyanobacterial endosymbiont into the host genome of an early ancestor of Archaeplastidae. Although Glaucophyta kept the genes of cyanobacterial origin, in red alga the aspartate pathway was not established and the kynurenine pathway remained active. In an early ancestor of green plants, 2) aspartate oxidase and 3) quinolinate synthase were substituted by horizontal gene transfers from nonphotosynthetic bacteria. 4) Gene transfer from an ancient green alga established quinolinate synthase (and possibly aspartate oxidase) in an early ancestor of Stramenopiles. 5) The aspartate oxidase gene observed in Stramenopiles was most likely acquired through horizontal gene transfer from a spirochaete or a halobacterium. This seems to be the most parsimonious explanation of the current evolutionary pattern of aspartate oxidases (fig. 3) and quinolinate synthases (fig. 4) in eukaryotes.

Our hypothesis that the aspartate pathway was introduced into eukaryotes by endosymbiotic gene transfer during the evolution of plastids is based on the observations that 1) the aspartate pathway is only detected in photosynthetic (or, in the case of Oomycota, closely related) eukaryotic clades, 2) in the glaucophyte *C. paradoxa* aspartate oxidase (fig. 3) and quinolinate synthase (fig. 4) seem to be of cyanobacterial origin, and 3) in green plants both enzymes are targeted to plastids (Katoh et al. 2006), as many gene products of endosymbiotic origin are. Although we think endosymbiotic acquisition of the aspartate pathway at the base of the Archaeplastidae is the most parsimonious explanation of the evolutionary trees presented here, alternative explanations cannot be ruled out. Not all photosynthetic eukaryotes use the aspartate pathway, such as red algae or the pelagophyte *Au. anophagefferens*, and not all eukaryotes using the aspartate pathway are photosynthetic, such as Oomycota. Yet Oomycota form a sister clade to several photosynthetic Stramenopiles, and probably descent from an ancestor with photosynthetic capacity (Cavalier-Smith and Chao 2006; Tyler et al. 2006). The aspartate oxidase and quinolinate synthase in *C. paradoxa* theoretically might have been acquired by horizontal—instead of endosymbiotic gene transfer, and with just one glaucophyte genome available, gene transfer from Cyanobacteria could be much more recent than indicated in figure 7. As the phylogeny of the plastid ancestor has not been resolved, and due to frequent gene transfer between bacteria never may be “resolved” (Dagan et al. 2013), evolutionary trees from single proteins, as in figures 3 and 4, do not allow to differentiate between endosymbiotic and horizontal gene transfer from Cyanobacteria into *C. paradoxa*. Yet with both, aspartate oxidase (fig. 3) and quinolinate synthase (fig. 4) originating from cyanobacteria, and quinolinate synthase being encoded in the cyanelle genome, endosymbiotic acquisition by an ancestor of *C. paradoxa* seems most parsimonious. Although many gene products with endosymbiotic ancestry are targeted to plastids (Timmis et al. 2004), not all plastid-targeted proteins are probably of endosymbiotic origin (Qiu et al. 2013). Therefore, the glaucophyte *C. paradoxa* and green plants could have acquired the aspartate pathway independent of each other, instead of a single endosymbiotic gene transfer at the base of the Archaeplastidae, as indicated in figure 7.

How some stramenopile clades acquired the aspartate pathway is not clear. Stramenopile aspartate oxidases form a sister group to aspartate oxidases from Spirochaeta and Halobacteriaeceae (fig. 3), whereas quinolinate synthases from Stramenopiles and Viridiplantae are sister groups neighboring Deltaproteobacteria and other Proteobacteria (fig. 4). We assume that aspartate oxidase was acquired by a Stramenopile ancestor through horizontal gene transfer from Sprochaeta or Halobacteriaceae, and that stramenopile quinolinate synthases originate from a gene transfer from Viridiplantae (fig. 7). However, due to the limited sequences in the evolutionary trees, we cannot determine the direction of potential gene transfers, and theoretically Viridiplantae might have acquired quinolinate synthase from Stramenopiles. A strong phylogenetic signal connecting Viridiplantae and Stramenopiles has been observed earlier, and gave rise to the hypothesis of a cryptic endosymbiosis between a heterotrophic ancestral stamenopile and an ancient green alga (Moustafa et al. 2009). However, both the extent of this phylogenetic signal and how it is to be interpreted are a matter of debate (Burki, Flegontov, et al. 2012; Deschamps and Moreira 2012; Woehle et al. 2011).

Both aspartate oxidase and nicotinate-nucleotide pyrophosphorylase (fig. 5) in Viridiplantae seem to originate from Bacteroidetes, even though for aspartate oxidase a proteobacterial origin cannot be ruled out (supplementary table S1, Supplementary Material online). Therefore, we assume that aspartate oxidase and nicotinate-nucleotide pyrophosphorylase were acquired in a single transfer event, possibly from a bacteroidetes encoding both enzymes in one operon. The quinolinate synthase in Viridiplantae was most likely acquired separately from a deltaproteobacterium.

### Evolutionary Origin of the Kynurenine Pathway in Eukaryotes

The evolutionary origin of the kynurenine pathway is unclear. The last common ancestor of all eukaryotes probably contained enzymes for the kynurenine pathway (see above; fig. 2), whereas archaea and most bacteria use the aspartate pathway. The patchy distribution of genes for the kynurenine pathway among different bacterial lineages gave rise to the hypothesis that some bacteria, especially Xanthomonadales and Flavobacteriales, have acquired these genes through horizontal transfer from eukaryotes (Lima et al. 2009). Yet with more and more bacterial genomes being sequenced and annotations improving, the kynurenine pathway is detected in species from more and more bacterial lineages. The Kyoto Encyclopedia of Genes and Genomes (Kanehisa et al. 2014) currently contains 91 bacterial genomes that contain genes encoding enzymes for at least four of the five catalyzed reactions of the kynurenine pathway. These are genomes from Gammaproteobacteria (23), Betaproteobacteria (13), Deltaproteobacteria (5), Alphaproteobacteria (1), Firmicutes (9), Actinobacteria (11), Acidobacteria (1), and Bacteroidetes (28). Evolutionary analyses of the enzymes catalyzing the kynurenine pathway (fig. 6 and supplementary figs. S3–S6, Supplementary Material online) currently do not support horizontal gene transfer from eukaryotes into Xanthomonadales, Flavobacteriales, or other bacterial lineages. Bacterial lineages are not nested within a clade of eukaryotic lineages showing the expected organismal phylogeny, as it is required to determine the direction of a gene transfer (Stanhope et al. 2001). This also holds for earlier evolutionary analyses (Lima et al. 2009), which do not show sequences from Xanthomonadales, Flavobacteriales, or other bacterial lineages embedded in a eukaryotic clade. It remains to be clarified whether the kynurenine pathway evolved in a very early eukaryote and spread to bacterial lineages (Lima et al. 2009), or whether it evolved in a bacterial lineage that contributed to the genome of the first eukaryotic organisms.

For some enzymes of the kynurenine pathway horizontal gene transfer from Bacteria or Archaea into eukaryotic genomes seems likely, such as the acquisition of tryprophan-2,3-dioxygenase by the excavate *N. gruberi* from Bacteria (or Archaea; fig. S3), and the acquisition of bacterial kynurenine-3-monooxygenase by different unicellular eukaryotes (fig. 6). In addition to a horizontal gene transfer from Bacteroidetes into an early ancestor of Amoebozoa, which had been reported earlier (Lima et al. 2009), kynurenine-3-monooxygenase has been acquired from Gammaproteobacteria by four unrelated marine eukaryotes, namely *P. marinus* (Alveolata), *S. arctica* (Ichthyosporea, Opist-hokonta), *Au. anophagefferens* (Pelagophycea, Strame-nopiles), and *Em. huxleyi* (Haptophyta). In this case it seems likely that a horizontal gene transfer from a marine gammaproteobacterium into a eukaryote, probably *P. marinus*, was followed be gene transfers from one unicellular marine eukaryote to another (fig. 7).

The pelagophyte *Au. anophagefferens* lacks genes for the aspartate pathway and instead encodes genes for the kynurenine pathway, whereas all other photosynthetic Stramenopiles appear to contain the aspartate pathway. This is especially puzzling, as the lineages of Pelagophyceae and brown algae (Phaeophyceae) are supposed to have split after the Oomycota (water molds or downy mildew) and diatoms (Bacillariophyceae) formed separate lineages (fig. 2) (Brown and Sorhannus 2010; Cavalier-Smith and Scoble 2013). Although the early diverging stramenopile clade Labyrinthulomycetes probably reflects the ancient state, that is, inherited the kynurenine pathway from the last common ancestor of all eukaryotes, this does not seem to be a valid explanation for *Au. anophagefferens* (fig. 7). Only the indoleamine-2,3-dioxygenases from *Au. anophagefferens* and Labyrinthulomycetes form a monophyletic group (supplementary fig. S4, Supplementary Material online). However, it is questionable, whether these putative stramenopile indoleamine-2,3-dioxygenases do indeed catalyze the first step of the kynurenine pathway (see above). Kynurenine-3-monooxygenases (catalyzing the third step of the kynurenine pathway) from *Au. anophagefferens* and Labyrinthulomycetes are not monophyletic (supplementary table S1, Supplementary Material online). *Aureococcus anophagefferens*, like other unicellular, marine eukaryotes, appears to have acquired a kynurenine-3-monooxygenase gene of gammaproteobacterial origin (fig. 6). An evolutionary tree of kynureninases (catalyzing the fourth step of the kynurenine pathway) does not show sequences from *Au. anophagefferens* and Labyrinthulomycetes as monophyletic either. However, there is no statistical support to rule out monophyly (supplementary table S1, Supplementary Material online). The 3-hydroxyanthranilate-3,4-dioxygenase (catalyzing the fifth step of the kynurenine pathway) from *Au. anophagefferens* is truncated preventing an evolutionary analysis. Moreover, the NAD^+^ synthase from *Au. anophagefferens* is of bacterial origin and not monophyletic with other eukaryotic sequences (supplementary fig. S2 and table S1, Supplementary Material online). In summary, the most parsimonious explanation seems that the common ancestor of oomycota, diatoms, brown algae, and the pelagophyte *Au. anophagefferens* acquired the aspartate pathway, whereas Labyrinthulomycetes kept the kynurenine pathway. After divergence from brown algae, the Pelagophyceae (or at least the lineage giving rise to *Au. anophagefferens*) then reacquired the kynurenine pathway. Although this might seem unlikely, it has been shown before that the assembly of an entire metabolic pathway through horizontal gene transfers into a eukaryotic genome has happened (Nedelcu et al. 2009; Pombert et al. 2012).

### Gene Transfers and the Evolution of Metabolic Pathways in Eukaryotes

Numerous gene transfers shaped de novo NAD^+^ biosynthesis in eukaryotes resulting in the acquisition of the aspartate pathway in different photosynthetic clades and the substitution of several enzymes of the kynurenine and the converged pathway. In figure 7, which summarizes these gene transfers, we intentionally did not differentiate between endosymbiotic and horizontal gene transfers because with currently available sequences these can hardly be discriminated. For the enzymes of the aspartate pathway in *C. paradoxa*, which are of cyanobacterial origin it seems reasonable to assume that they were acquired through endosymbiotic gene transfer. The same holds for nicotinate-nucleotide pyrophosphorylase in *B. natans*, which probably was acquired from green plants during the secondary endosymbiosis giving rise to plastids in Chlorarachniophyta. However, horizontal gene transfer cannot be ruled out in either case as an alternative explanation. On the other hand, the enzymes of the aspartate pathway in Viridiplantae seem to originate from nonphotosynthetic bacteria making an acquisition through endosymbiotic gene transfer during plastid evolution unlikely. However, the plastid ancestor, while still a free-living cyanobacterium, might have acquired these two genes encoding the aspartate pathway through horizontal gene transfer from nonphotosynthetic bacteria, followed by endosymbiotic gene transfer of these two genes during plastid evolution. Neither has the phylogenetic origin of plastids within Cyanobacteria been resolved nor is the acquisition of hundreds of genes in green plants originating from other prokaryotes well understood (Dagan et al. 2013).

Regardless whether endosymbiotic or horizontal the relative large number of gene transfers shaping NAD^+^ biosynthesis in eukaryotes is unexpected. De novo NAD^+^ biosynthesis is essential, and lack of just one enzyme is usually lethal (Katoh et al. 2006; Lin et al. 2010; Panozzo et al. 2002). Therefore, one might expect high conservation of de novo NAD^+^ biosynthesis. In contrast, our evolutionary analyses show that entire pathways were swapped (kynurenine vs. aspartate pathway), and enzymes were substituted by orthologs from bacteria through horizontal gene transfer. Horizontal gene transfer is very common in Bacteria and Archaea (Koonin et al. 2001; Treangen and Rocha 2011) and there are indications that adaptive evolution of bacterial metabolic networks was largely driven by horizontal gene transfers (Pal et al. 2005). Systematic screens of eukaryotic genomes for genes originating from horizontal transfer identified many genes encoding enzymes (Schönknecht et al. 2014; Whitaker et al. 2009), and essential metabolic pathways, such as isoprenoid biosynthesis (Lange et al. 2000), heme biosynthesis (Oborník and Green 2005), or the shikimate pathway (Richards et al. 2006) have been shown to be encoded by a mosaic of genes inherited vertically and acquired through endosymbiotic or horizontal gene transfers. In addition to de novo NAD^+^ biosynthesis, salvage biosynthesis of NAD^+^ starting with nicotinamide was shaped by horizontal gene transfers as well. Phylogenetic analyses of the two nicotinamide-metabolizing enzymes nicotinamide phosphoribosyltransferase (EC 2.4.2.12) and nicotinamidase (EC 3.5.1.19) show clear deviations from established organismal phylogenies, indicating several instances of horizontal gene transfer (Gazzaniga et al. 2009). For nicotinamide phosphoribosyltransferase, there are evidences for virus- and plasmid-mediated gene transfers (Gazzaniga et al. 2009). Gene transfers seem to have contributed significantly to the evolution of metabolic pathways in eukaryotes, as shown here for de novo NAD^+^ biosynthesis.

## Supplementary Material

Supplementary figures S1–S6 and table S1 are available at *Genome Biology and Evolution* online (http://www.gbe.oxfordjournals.org/).

Supplementary Data
