# Supplementary material for: Gene Transfers Shaped the Evolution of De Novo NAD+ Biosynthesis in Eukaryotes
Source: Genome Biol Evol. 2014 Aug 27;6(9):2335–49. doi: 10.1093/gbe/evu185 (PMC4217691; doi:10.1093/gbe/evu185)
Supplement: Supplementary Data [file supp_6_9_2335__index.html]

Gene transfers shaped the evolution of de novo NAD+ biosynthesis in eukaryotes — Gene Transfers Shaped the Evolution of De Novo NAD+ Biosynthesis in Eukaryotes — Supplementary Data 

# Gene Transfers Shaped the Evolution of De Novo NAD+ Biosynthesis in Eukaryotes

## Supplementary Data

files

**Files in this Data Supplement:**

- Supplementary Data - pdf file
