# Supplementary material for: Gene Transfers Shaped the Evolution of De Novo NAD+ Biosynthesis in Eukaryotes
Source: Genome Biol Evol. 2014 Aug 27;6(9):2335–49. doi: 10.1093/gbe/evu185 (PMC4217691; doi:10.1093/gbe/evu185)
Supplement: Supplementary Data [file supp_evu185_NAD_Biosynth_Suppl.pdf]

Supplementary Material

Gene transfers shaped the evolution of *de novo* NAD^+ biosynthesis in eukaryotes

Chad M. Ternes and Gerald Schönknecht*

Department of Botany, Oklahoma State University, Stillwater, OK 74078, USA

*Author for Correspondence: Gerald Schönknecht, Department of Botany, Oklahoma State University, phone: +1-405-744-5549, fax: +1-405-744-1797, gerald.schoenknecht@okstate.edu

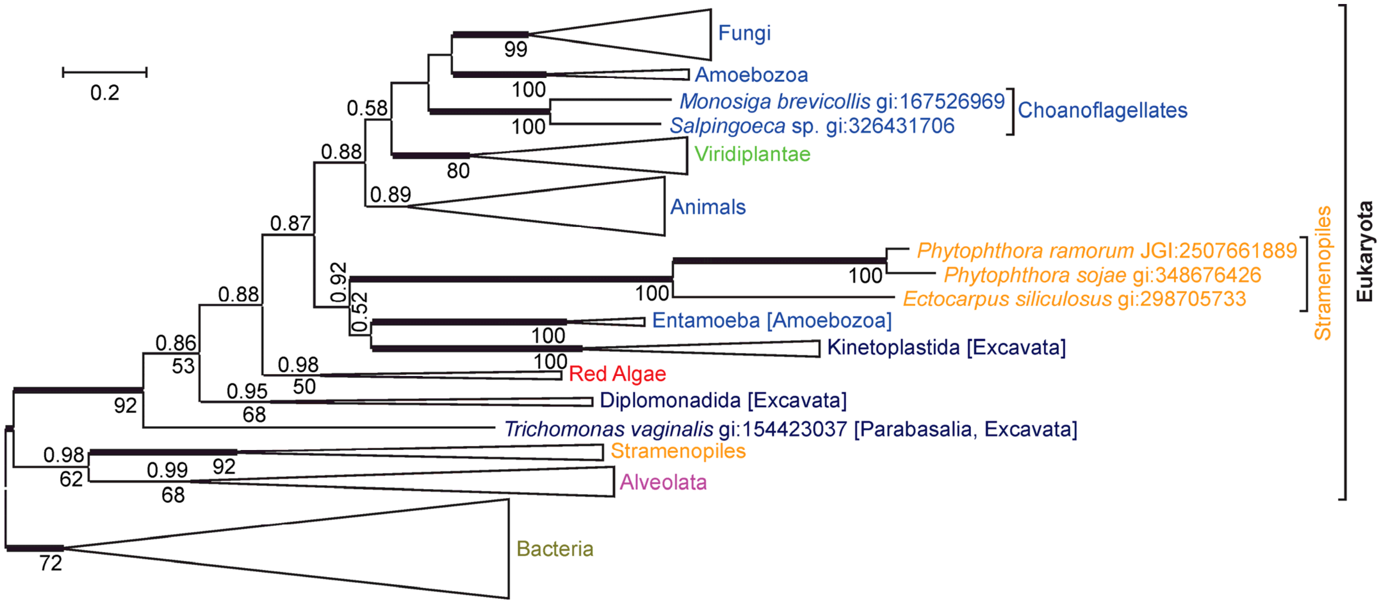

FIG. S1. An evolutionary tree for nicotinate-nucleotide adenylyltransferase shows the expected separation of bacterial and eukaryotic sequences. The unrooted Bayesian tree was generated using the VT+G model of protein evolution with posterior probabilities above the branches and PhyML bootstrap values (LG+G) displayed as percentages below the branches. A score of 3 to 9 instead of 5 to 9 was used to extract multiple sequence alignments. Thickened horizontal lines represent 1.0 Bayesian posterior probability. Larger clades have been collapsed for presentation and size is indicative of the number of taxa within the clade. Color coding of major phylogenetic groups as in fig. 2 (Bacteria, Unikonta, Excavata, Stramenopiles, Alveolata, Viridiplantae, Red Algae). Scale bar represents 0.2 substitutions per site.

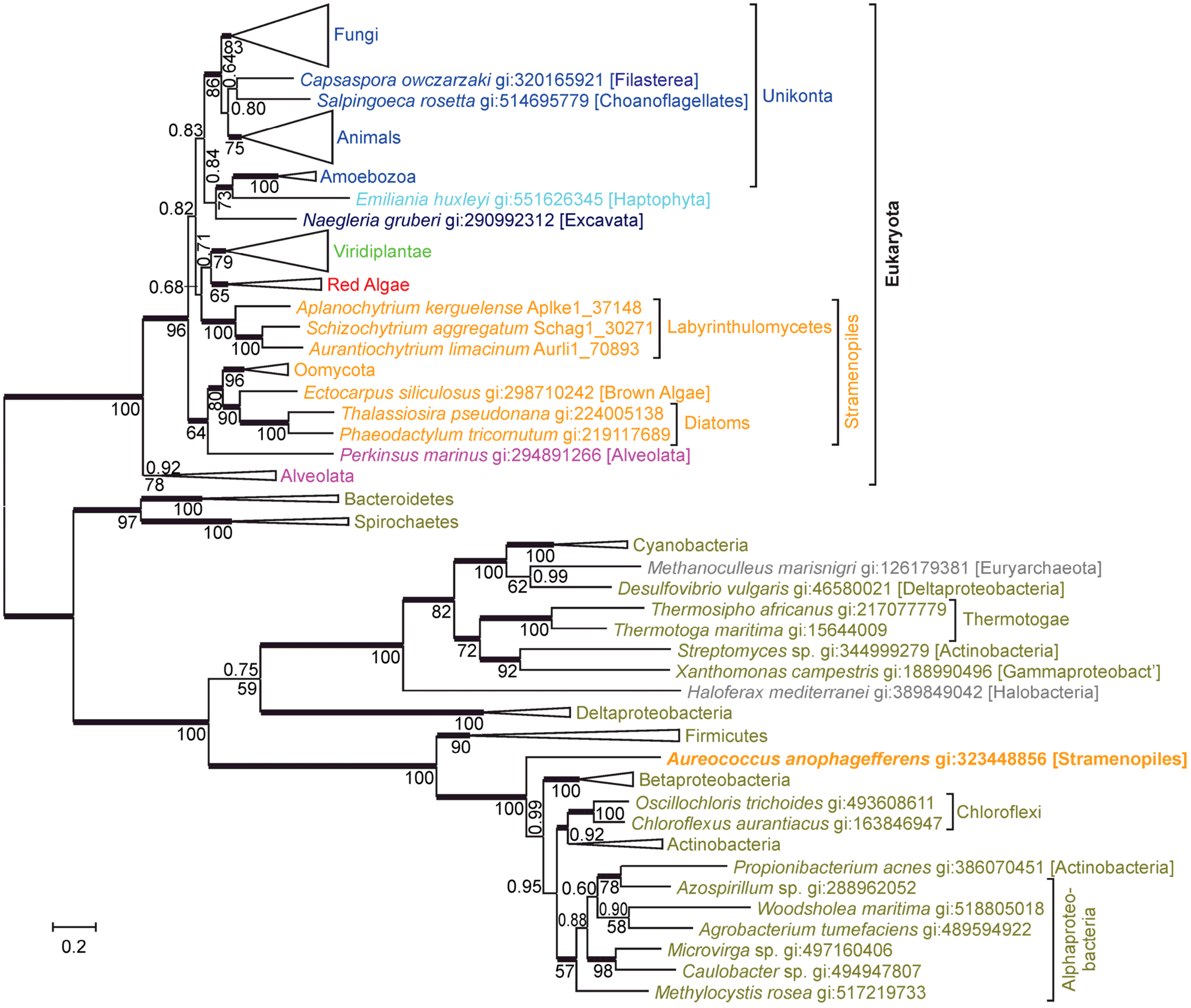

FIG. S2. An evolutionary tree for NAD⁺ synthases indicates that *A. anophagefferens* acquired a gene via horizontal gene transfer from a bacterium. The unrooted Bayesian tree shows posterior probabilities above the branches and PhyML bootstrap values (LG+I+G+F) below the branches. Thickened horizontal lines represent 1.0 Bayesian posterior probability. Trees with significantly lower log-likelihoods were obtained when monophyly was enforced for all sequences from Eukaryota, or from Bacteria (supplementary table 1, Supplementary Material online).

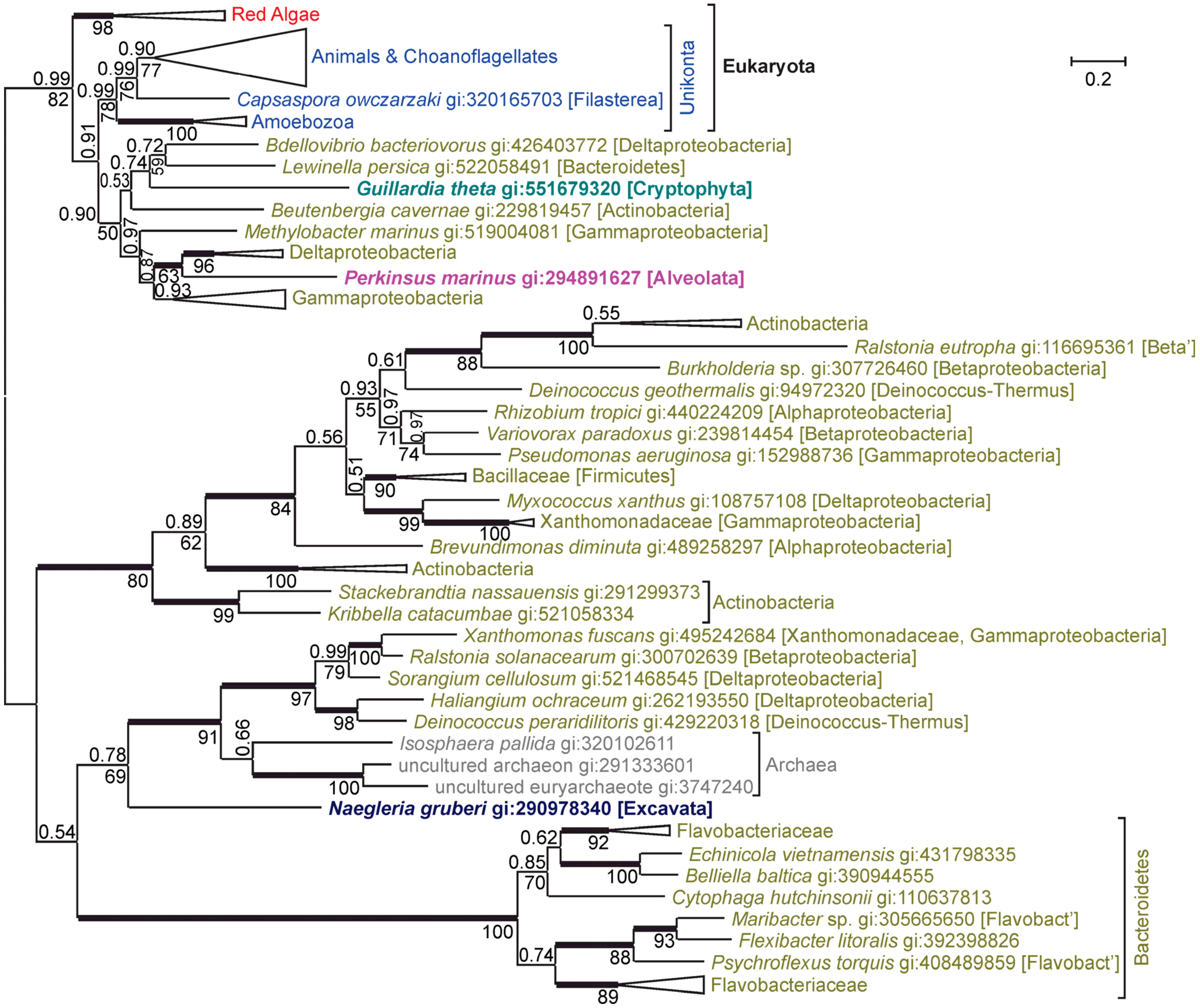

FIG S3. An evolutionary tree for tryprophan-2,3-dioxygenase indicates that *N. gruberi* acquired a gene via horizontal gene transfer. The unrooted Bayesian tree shows posterior probabilities above the branches and PhyML bootstrap values below the branches. A score of 3 to 9 instead of 5 to 9 was used to extract multiple sequence alignments. Thickened horizontal lines represent 1.0 Bayesian posterior probability. A tree with practically identical log-likelihoods was obtained when monophyly was enforced for all sequences from Unikonta plus red algae. Trees with significantly lower log-likelihoods were obtained when monophyly was enforced for all sequences from Eukaryota, or from Unikonta plus red algae plus *N. gruberi* (supplementary table 1, Supplementary Material online).

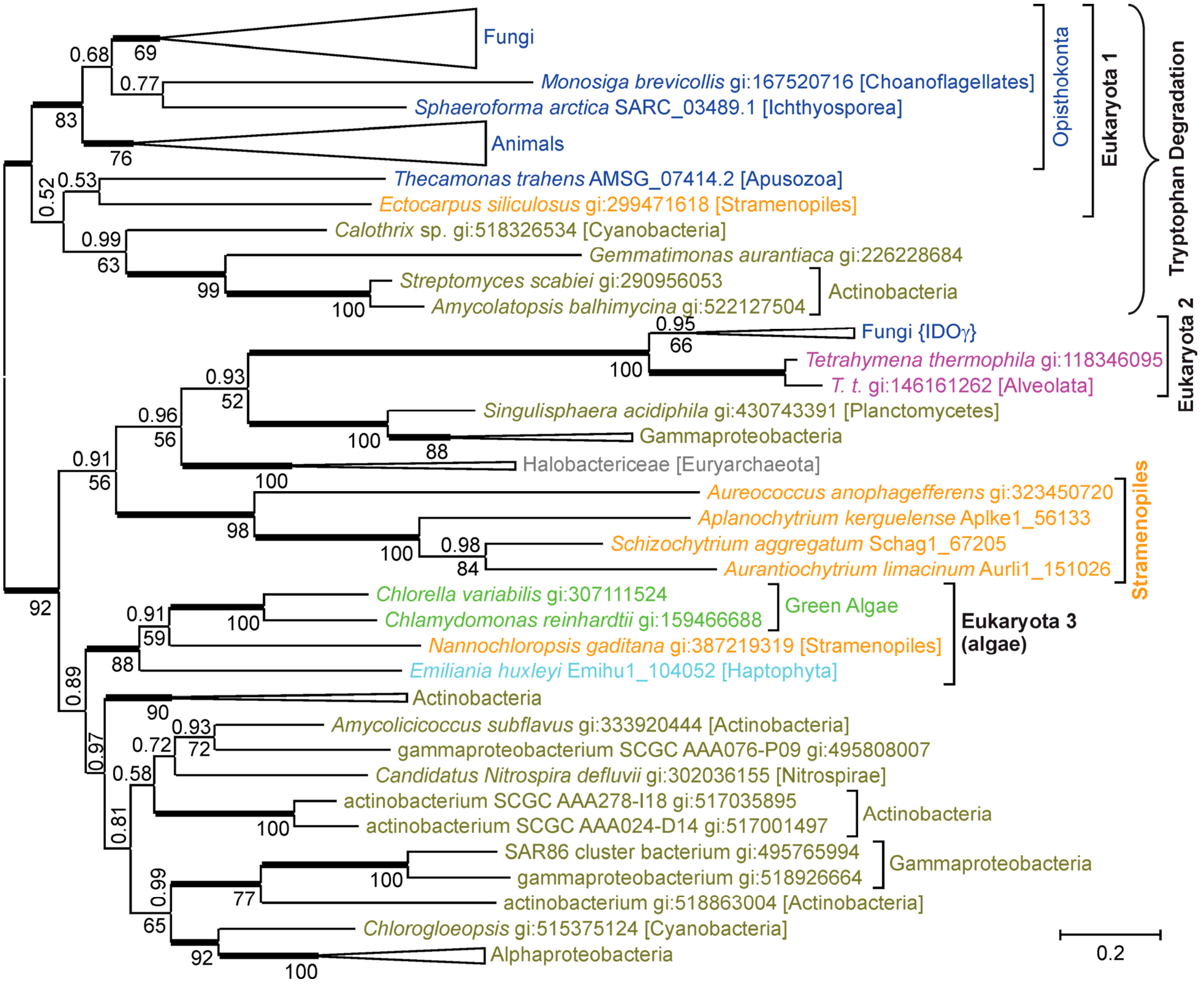

FIG. S4. An evolutionary tree for indoleamine-2,3-dioxygenase shows a complex mixture of bacterial and eukaryotic sequences. The unrooted Bayesian tree shows posterior probabilities above the branches and PhyML bootstrap values below the branches. Thickened horizontal lines represent 1.0 Bayesian posterior probability. Trees with significantly lower log-likelihoods were obtained when monophyly was enforced for all sequences from Eukaryota, or from Bacteria (supplementary table 1, Supplementary Material online). Curly brackets refer to enzymatic activity; only enzymes from the upper branch show reaction rates as expected for effective tryptophan degradation, while fungal indoleamine-2,3-dioxygenase γ (IDOγ) and some bacterial enzymes in the lower two branches have very low reaction rates (Yuasa and Ball 2012; Yuasa, et al. 2011).

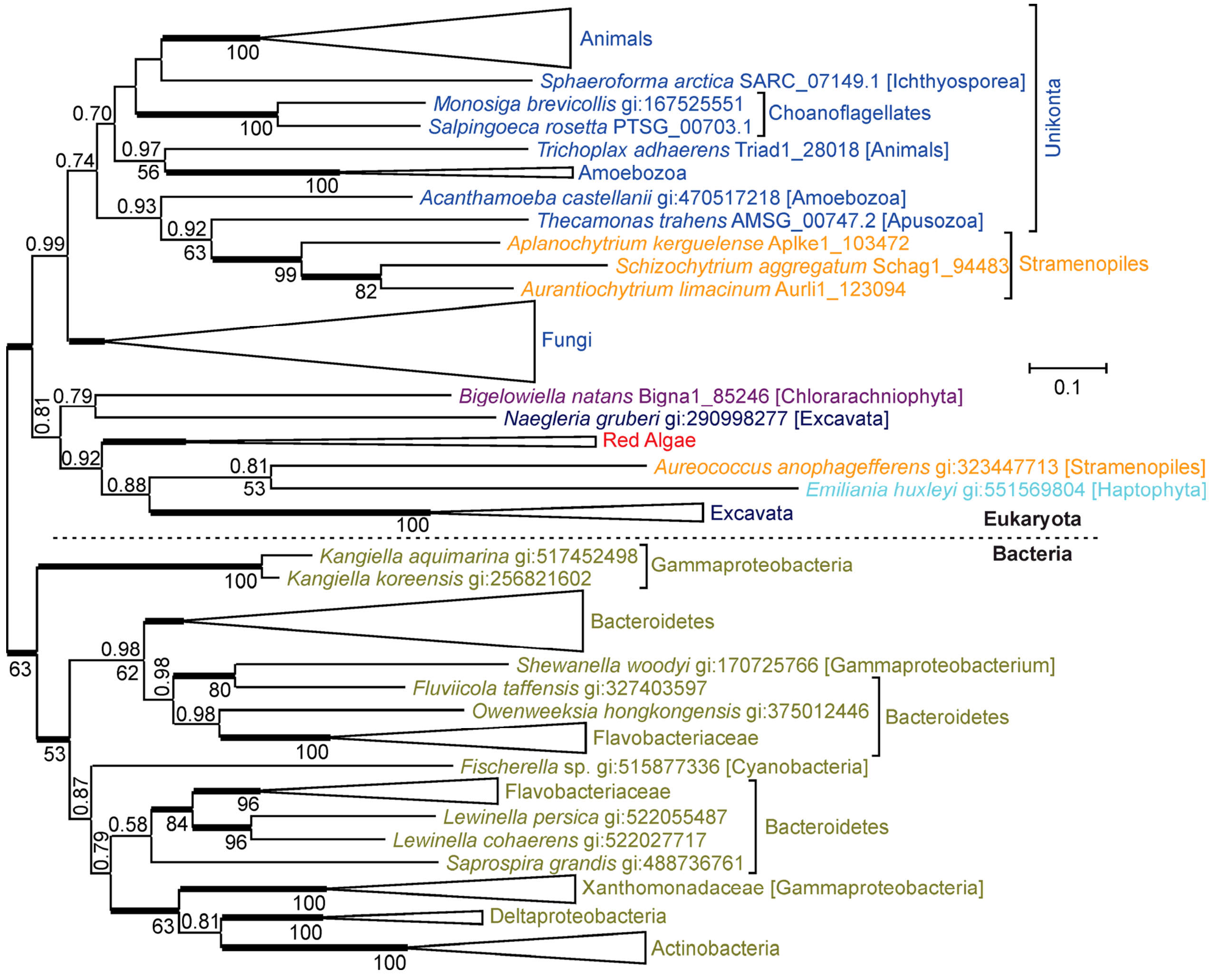

FIG. S5. An evolutionary tree of kynureninases shows the expected separation of eukaryotic and bacterial sequences. The unrooted Bayesian tree shows posterior probabilities above the branches and PhyML bootstrap values below the branches. Thickened horizontal lines represent 1.0 Bayesian posterior probability.

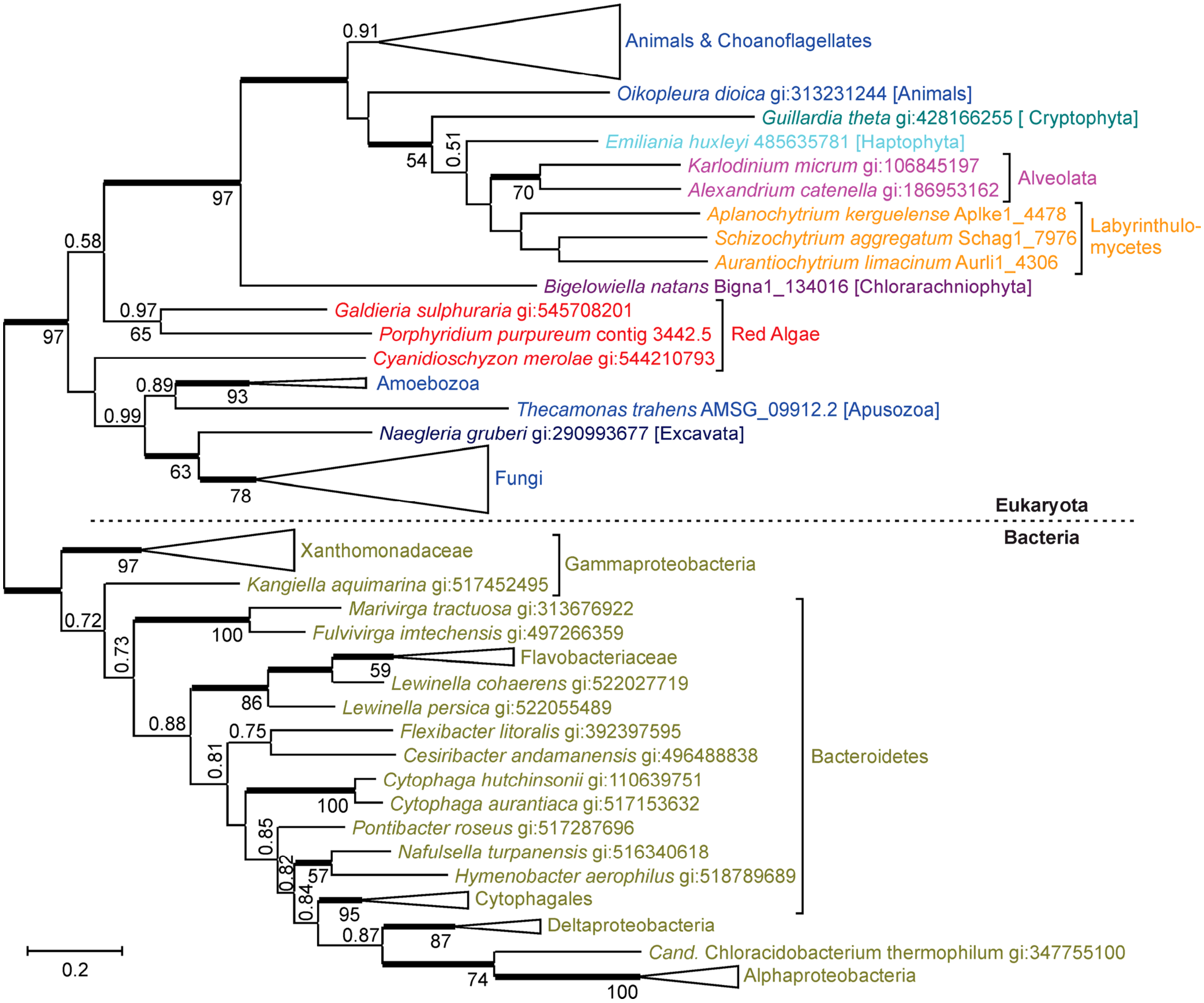

FIG. S6. An evolutionary tree of 3-hydroxyanthranilate-3,4-dioxygenases shows the expected separation of eukaryotic and bacterial sequences. The unrooted Bayesian tree shows posterior probabilities above the branches and PhyML bootstrap values below the branches. Thickened horizontal lines represent 1.0 Bayesian posterior probability. The sequence from *Aureococcus anophageferens* was omitted from an evolutionary analysis because it is incomplete.

Table S1. Bayesian and Maximum Likelihood analyses of constraint trees. From left to right, clades for which protein sequences were forced to be monophyletic, harmonic mean (*H*) of log-likelihood values from stationary phase of Bayesian analysis as calculated by Tracer (http://beast.bio.ed.ac.uk/Tracer), difference between harmonic means of unconstrained and constrained tree (Δ*H*), Akaike's information criterion through Markov chain Monte Carlo (AICM) as calculated by Tracer, difference in AICM between unconstrained and constrained tree (ΔAICM), log-likelihood (log *L*) of best maximum likelihood tree, difference between log-likelihoods (Δlog *L*) of unconstrained and constrained tree, *p*-values from approximately unbiased (AU) test and from Kishino-Hasegawa (KH) test as calculated by CONSEL (Shimodaira 2002; Shimodaira and Hasegawa 2001). QS, quinolinate synthase, AO, L-aspartate oxidase. Δ*H*, ΔAICM, and *p*-values indicating no significant difference in italics and reduced font size.

Monophyletic Constraint	Bayesian				Maximum Likelihood			
	H	**Δ*H***	**AICM**	**ΔAICM**	**log *L***	**Δlog *L***	**AU**	**KH**
					Aspartate Oxidase			
Eukaryota	-51141	323	102430	-634	-50703	314	3.0E-06	0
Viridiplantae & *C. paradoxa*	-51048	230	102249	-453	-50612	224	2.0E-05	0
Viridiplantae & Stramenopiles [as in QS tree]	-50952	135	102047	-251	-50507	118	4.0E-06	0.0001
Stramenopiles & *C. paradoxa*	-50979	161	102109	-312	-50547	158	7.0E-06	9.0E-06
Bacteria	-51153	336	102460	-664	-50717	329	3E-254	0
Archaea	-51148	330	102452	-656	-50434	46.2	*0.054*	*0.058*
Viridiplantae & Proteobacteria [as in QS tree]	-50943	126	102043	-246	-50397	8.2	*0.32*	*0.33*
Stramenopiles & Proteobacteria [as in QS tree]	-51020	202	102207	-411	-50482	93.7	4.0E-05	0
Stramenopiles & Halobacteriaceae	-50827	*9.1*	101802	*-5.8*	-50391	2.8	*0.274*	*0.29*
					Quinolinate Synthase			
Eukaryota	-29507	344	59159	-684	-29175	332	1.0E-92	0
Viridiplantae & *C. paradoxa*	-29552	389	59274	-800	-29219	375	9E-121	0

Bacteria	-29842	679	59838	-1363	-29465	621	3.0E-29	0
Archaea	-29282	119	58714	-239	-28965	121	1.0E-06	0
Viridiplantae & Bacteriodetes [as in AO tree]	-29577	414	59312	-838	-29247	404	4E-116	0
Stramenopiles & Halobacteriaceae [as in AO tree]	-29407	244	58975	-501	-29083	240	2.0E-14	0
Stramenopiles & Spirochaetes [as in AO tree]	-29419	256	59000	-525	-29088	245	3.0E-12	0
				Nicotinate-Nucleotide Pyrophosphorylase				
Eukaryota	-38680	61.6	77583	-104	-38283	59.9	0.019	0.018
Viridiplantae & *B. natans*	-38630	*11.5*	77485	*-5.5*	-38224	1.1	*0.474*	*0.476*
Viridiplantae & Cyanobacteria	-38654	*36.1*	77555	-75.7	-38250	27.8	*0.099*	*0.092*
Viridiplantae & Red Algae	-38851	233	77943	-463	-38443	220	4.0E-06	0
Viridiplantae & Stramenopiles [as in QS tree]	-38857	238	77958	-478	-38429	206	5.0E-15	0
B. natans & Stramenopiles	-38783	165	77803	-323	-38381	159	7.0E-08	0
Bacteria	-38642	*23.5*	77519	*-39.3*	-38253	29.9	*0.077*	*0.072*
Opisthokonta	-38641	*22.9*	77524	*-43.9*	-38235	11.8	*0.173*	*0.172*
Unikonta	-38637	*19.3*	77496	*-16.8*	-38235	11.8	*0.172*	*0.174*
Red Algae	-38624	*5.7*	77478	*1.2*	-38231	8.5	*0.232*	*0.223*
Stramenopiles	-38633	*15.0*	77478	*2.1*	-38226	3.4	*0.417*	*0.424*
Stramenopiles & Halobacteriaceae [as in AO tree]	-38686	67.9	77623	-143	-38273	49.9	*0.057*	*0.062*
				Nicotinate-Nucleotide Adenylyltransferase				
Amoebozoa	-41091	*15.5*	82552	*-24.9*	-40058	20.2	0.016	0.030
Excavata	-41101	*25.9*	82577	*-50.6*	-40051	13	*0.310*	*0.307*
Stramenopiles	-41135	59.1	82601	*-74.5*	-40086	48.2	0.004	0.006
Unikonta	-41089	*13.1*	82537	*-10.7*	-40053	15.3	*0.191*	*0.196*
Viridiplantae & Red Algae	-41087	*11.4*	82550	*-23.3*	-40048	10.5	*0.367*	*0.350*

				NAD$^+$ Synthase				
Eukaryota	-101181	222	202620	-458	-100249	227	0	0
Bacteria	-101692	733	203653	-1490	-100755	733	0	0
Unikonta	-100973	*13.9*	202195	*-32.0*	-100040	18.3	*0.209*	*0.190*
Stramenopiles (without *A. anophagefferens*)	-101135	176	202510	-348	-100040	17.9	*0.204*	*0.203*
Alveolata	-101000	40.8	202213	*-50.1*	-100046	24.1	*0.143*	*0.138*
				Tryptophan 2,3-Dioxygenase				
Eukaryotes (all)	-40116	68.6	80399	-150	-39838	73.6	0.013	0.011
Unikonta & Red Algae	-40038	*-8.7*	80267	*-17.2*	-39769	5.1	*0.370*	*0.357*
Unikonta & Red Algae & Naegleria	-40095	48.2	80374	-125	-39817	52.6	0.001	0.001
Unikonta & Red Algae & Perkinsus	-40057	*10.5*	80300	*-50.2*	-39791	26.6	*0.077*	*0.081*
Unikonta & Red Algae & Guillardia	-40044	*-2.7*	80257	*-7.1*	-39772	8.4	*0.283*	*0.284*
Perkinsus & Guillardia	-40051	*4.0*	80282	*-31.8*	-39781	16.7	*0.054*	*0.054*
Bacteria	-40206	159	80596	-346	-39927	163	6.0E-18	0
Gammaproteobacteria	-40772	726	81733	-1483	-40492	728	9.0E-70	0
Deltaproteobacteria	-40771	724	81690	-1440	-40469	705	2.0E-05	0
				Indoleamine 2,3-Dioxygenase				
Eukaryota	-44187	82.5	88534	-166	-43890	78.8	9.0E-05	0.001
Bacteria	-44198	93.2	88541	-173	-43913	102	6.0E-08	0
Stramenopiles & Eukaryota 2	-44112	*7.9*	88374	*-6.3*	-43813	1.8	*0.408*	*0.414*
Stramenopiles & Eukaryota 2 & Eukaryota 1	-44150	45.2	88459	-91.2	-43851	40	0.026	0.030
Stramenopiles & Eukaryota 2 & Eukaryota 3	-44135	*30.9*	88422	*-54.2*	-43836	24.7	0.036	0.037
Eukaryota 1 & Eukaryota 3	-44171	66.7	88497	-129	-43873	62	0.0001	0.001
E. siliculosus & Eukaryota 3 (algae)	-44146	41.2	88451	-83.0	-43853	41.8	0.001	0.001

E. siliculosus & Stramenopiles	-44120	*15.6*	88398	*-30.2*	-43828	16.9	*0.134*	*0.136*
Stramenopiles (from all three branches)	-44164	59.4	88490	-122	-43871	59.7	0.0001	0
Fungi	-44424	319	89004	-636	-44118	307	4.0E-09	0
					Kynurenine-3-Monooxygenase			
Eukaryotes (all)	-581589	41.0	116502	-94.6	-57736	45.3	0.015	0.02
Unikonta without *S. artica*	-581823	64.7	116546	-139.3	-57755	64.2	0.032	0.04
Opisthokonta	-58230	112.4	116659	-252.1	-57819	128.0	2.0E-06	0
SAR & Haptophyta	-58250	131.8	116712	-304.5	-57830	139.1	3.0E-79	0
Stramenopiles	-58438	319.7	117095	-687.5	-58009	318.5	2.0E-38	0
Eukaryota 1 & Eukaryota 2 (marine)	-58138	*20.1*	116470	*-62.7*	-57723	32.4	0.003	0.013
Amoebozoa & Eukaryota 2 (marine)	-58138	*20.5*	116443	*-35.5*	-57704	13.3	*0.096*	*0.107*
					Kynureninase			
Unikonta	-73137	*11.5*	146577	*-38.6*	-72570	9.6	*0.155*	*0.160*
Stramenopiles	-73152	*27.2*	146574	*-35.3*	-72588	27.7	*0.103*	*0.102*
Excavata	-73131	*5.4*	146546	*-7.4*	-72564	3	*0.446*	*0.434*
Stramenopiles & *B. natans* (SAR)	-73152	*27.2*	146574	*-35.3*	-72596	34.9	*0.070*	*0.072*
					3-Hydroxyanthranilate-3,4-Dioxygenase			
Unikonta	-25839	111	51894	-189	-25578	105	4.0E-08	0.0003
Opisthokonta	-25843	115	51926	-221	-25584	111	3.0E-44	0
Red Algae	-25737	*8.8*	51713	*-8.2*	-25475	1.4	*0.161*	*0.226*
Stramenopiles & Alveolates & *B. natans* (SAR)	-25755	*26.6*	51742	*-36.9*	-25497	23.7	0.046	*0.051*

References

Shimodaira H. 2002. An approximately unbiased test of phylogenetic tree selection. Syst Biol 51: 492-508.

Shimodaira H, Hasegawa M. 2001. CONSEL: for assessing the confidence of phylogenetic tree selection. Bioinformatics 17: 1246-1247.

Yuasa HJ, Ball HJ. 2012. The evolution of three types of indoleamine 2,3 dioxygenases in fungi with distinct molecular and biochemical characteristics. Gene 504: 64-74.

Yuasa HJ, Ushigoe A, Ball HJ. 2011. Molecular evolution of bacterial indoleamine 2,3-dioxygenase. Gene 485: 22-31.
